# Visual attention mechanism and support vector machine based automatic image annotation

**DOI:** 10.1371/journal.pone.0206971

**Published:** 2018-11-06

**Authors:** Zhangang Hao, Hongwei Ge, Long Wang

**Affiliations:** 1 School of Business Administration, Shandong Technology and Business University, Yantai, Shandong, China; 2 School of Computer Science and Technology, Dalian University of Technology, Dalian, Liaoning, China; North Shore Long Island Jewish Health System, UNITED STATES

## Abstract

Automatic image annotation not only has the efficiency of text-based image retrieval but also achieves the accuracy of content-based image retrieval. Users of annotated images can locate images they want to search by providing keywords. Currently most automatic image annotation algorithms do not consider the relative importance of each region in the image, and some algorithms extract the image features as a whole. This makes it difficult for annotation words to reflect salient versus non-salient areas of the image. Users searching for images are usually only interested in the salient areas. We propose an algorithm that integrates a visual attention mechanism with image annotation. A preprocessing step divides the image into two parts, the salient regions and everything else, and the annotation step places a greater weight on the salient region. When the image is annotated, words relating to the salient region are given first. The support vector machine uses particle swarm optimization to annotate the images automatically. Experimental results show the effectiveness of the proposed algorithm.

## 1. Introduction

With the development of digital and Internet technologies, there are massive numbers of digital images on the Internet. It is also becoming harder and harder for users to find these images accurately and quickly. The dramatic increase in the number of images on the Internet combined with the subjectivity, uncertainty and laboriousness of manual annotation has led to a gradual failure to apply text-based image retrieval (TBIR) to large-scale image retrieval [1]. In comparison, content-based image retrieval (CBIR) increases the amount of effort from users, often requiring them to provide initial images as input for the search. So, CBIR has not been used widely [2–5]. Image retrieval process can be indirectly changed into text retrieval by automatic image annotation [6–9]. That is, users need to provide only the query keywords, with the system returning the images associated with the keywords, which is in line with most users’ current habits. Automatic image annotation has become an important research topic in the field of image retrieval. As long as the image can be accurately annotated, good results can be returned to the user.

Segmenting image is very important for automatic image annotation, greatly influencing the annotation results. Most existing automatic image annotation algorithms do not consider the relative importance of different parts of the image to the user when segmenting the image. Other annotation algorithms do not segment the image at all but extract the whole image as a feature. In this paper, visual attention mechanism is introduced into the annotation process. During pre-processing, the image is divided into salient and non-salient regions. During annotation, salient regions of a image are weighted to give them higher priority. This weighting indirectly enhances the performance of image retrieval (because search keywords often reflect the users’ interest only in the salient regions) of the image.

## 2. Related work

Since this paper uses a support vector machine (SVM) for automatic image annotation, we divided the existing automatic image annotation algorithms into two categories: automatic image annotation using a support vector machines and automatic annotation based on other methods.

**Automatic image annotation based on SVM**: Cusano et al. proposed a classification system using multi-class support vector machines for automatic image annotation which can be used for large-scale video and image management [10]. Gao et al. proposed a hierarchical boosting algorithm to enhance SVM image classifier training for image annotation [11]. Ommasi et al. proposed SVM-based methods to annotate medical images [12]. Verma proposed a new loss function to make the SVM more robust, solving three problems in annotation: incomplete labels, fuzzy labels, structural overlap [13]. Wu et al. proposed a feature selection method, constructing a multi-class classifier using weighted probabilities and an improved SVM [14]. Yu et al. proposed an automatic image annotation algorithm based on a superpixel bag of words model and an SVM classifier [15]. Majidpour et al. used principal components analysis (PCA) to reduce the number of color features followed by an SVM for classification [16]. Jin et al. proposed a multi-label image automatic labeling framework using an improved multi-kernel learning SVM [17]. Hao et al. proposed an automatic image annotation method based on particle swarm optimization (PSO) and support vector clustering (SVC). They use PSO to optimize the SVC for automatic image annotation [9].

**Automatic image annotation based on other methods**: For image annotation, Makadia et al. proposed a new baseline technique that uses low-level image features and a simple distance algorithm to find the nearest neighbor of a given image [18]. Weston et al. proposed an automatic annotation algorithm for large-scale image datasets that uses simultaneous learning [19]. Liu et al. proposed a multiview Hessian regularization (mHR) algorithm applied to kernel least squares and SVMs for image annotation [20]. Employing jaccard similarities, Johnson et al. used multiple non-parametric image metadata to identify neighbors of related images followed by a deep neural network to annotate images [21]. Tariq et al. proposed a strategy that performed a tensor analysis of new images to evaluate the context, and then combined the evaluated content and the image content to label images [22]. In order to solve the problem with incomplete labels for many training image datasets, Wu et al. used incomplete label data to train classifiers [23]. Shin et al. proposed a deep learning model to detect disease within medical images [24]. Jing et al. proposed a new multi-label learning method that integrates multi-label dictionary learning with embedding of some of the same tags [25]. Rad et al. proposed a new automatic image annotation method based on non-negative matrix factorization [26]. Liu et al. used kernel logistic regression to annotate network images automatically [27]. Choi et al. annotated images by analyzing images and image-related text [28]. Uricchio et al. proposed a label propagation framework based on kernel canonical correlation analysis[29]. Chang et al. performed rapid image annotation using brain state decoding and visual pattern mining [30].

From the literature review, we can see that although there are many works for automatically annotating images, these methods rarely consider the relative importance of each part of the image to users when segmenting image. Therefore, this paper introduces a visual attention mechanism to solve this problem. We use PSO to optimize an SVM, which can improve the accuracy of image annotation.

## 3. Histogram-based contrast method

The histogram-based contrast method (HC) uses the image global histogram features to extract salient regions of images, in a bottom-up, data-driven approach. The generally accepted theory is that cortical cells of the human brain are genetically predisposed to respond to high contrast stimulation. Based on this theory, a high-resolution, global saliency map contrast analysis approach is proposed:

1. Based on the global contrast method, large-scale objects will be separated from the surrounding environment. This method outperforms a local contrast method that produces significant values only at or near the edge of the object.
2. Global considerations will assign similar salient values to similar regions, consistently highlighting the entire salient object.
3. The significance of the area will depend mainly on the area around it, with distant areas having less impact.
4. The saliency map should be generated simply and quickly to accommodate large-scale image processing and efficient image classification and retrieval.

An HC-Map based on histogram contrast will be used to assign pixel significant values based on color differences from other pixels, producing an image saliency map with full resolution.

### 3.1. Pixel salient value

Based on the sensitivity of a visual system to the visual signal contrast in biological vision, a histogram-based contrast method is proposed to define the saliency value of each pixel in the image. Specifically, the saliency value calculation for each pixel is obtained by calculating its contrast with every other pixel, that is, the salient value of the pixel in the image is defined as formula (1).

$$S(I_{k})=\sum_{\forall I_{i}\in I}D(I_{k},I_{i})$$ (1)

where *D*(*I*_*k*_, *I*_*i*_) is the distance of pixel *I*_*k*_ and *I*_*i*_ in the Lab color space. Formula (1) can be expanded to formula (2).

$$S(I_{k})=D(I_{k},I_{1})+D(I_{k},I_{2})+\ldots +D(I_{k},I_{N})$$ (2)

where *N* is the total number of pixels in the image *I*.

With these definitions, it is easy to see that pixels with the same color value have the same salient value because the spatial relationship of the pixels is ignored. Then, formula (2) can be rearranged so that the values having the same color are grouped into one class with the saliency value of each color value obtained as a result.

$$S(I_{k})=S(c_{l})=\sum_{j=1}^{n}f_{i}•D(c_{l},c_{j})$$ (3)

where *c*_*l*_ is the color value of the pixel *I*_*k*_; *n* is the number of colors contained in the image; *f*_*i*_ is the probability of *c*_*j*_ in the image.

### 3.2. Acceleration based on histogram

The time complexity of calculating the salient value of each pixel in the image by formula (1) is *O*(*N*^2^), which has very high computational cost for a medium sized image. Eq (3) is equivalent to Eq (2), but its time complexity is reduced to *O*(*N*) + *O*(*n*^2^), which means that if *O*(*n*^2^) ≤ *O*(*N*), the time complexity is *O*(*N*). Therefore, the reduction in the number of pixel colors becomes key. If the RGB color space is used, the maximum number of possible colors per pixel is 256^3^, far greater than the total number of pixels.

In order to reduce the total number of colors, we can reduce the number of colors per channel to 12 from 256 and the total number of colors can be reduced to 12^3^ = 1728. Colors in actual use are only a small percentage of the total color space, and colors that appear less frequently can be discarded to further reduce the number of colors. By selecting the colors that appear at high frequencies, the number of pixels in these colors is guaranteed to account for 95% of the whole image.

### 3.3. Smoothing color space

**A**lthough quantifying colors and extracting colors with high frequency can effectively improve the computational efficiency, there are some shortcomings in the quantification itself. Some similar colors may be incorrectly quantized to colors with different values. To reduce the effect of this random noise on the calculation of saliency values, the use of a smoothing process for each color will improve saliency values. The specific operation replaces the saliency value of each color with a weighted average value of the saliency values of similar colors (measured in terms of distance in the Lab color space). Pick the *m* = *n*/4 nearest color to improve the color *c* saliency value.

$$S^{\prime }(c)=\frac{1}{(m-1)T}\sum_{i=1}^{m}\left(T-D(c,c_{i})\right)S(c_{i})$$ (4)

where $T=\sum_{i=1}^{m}D(c,c_{i})$ is the sum of the distance of the color *c* from its nearest neighbor *m*, and the normalization factor is (*m* − 1)*T*.

## 4. Automatic annotation based on visual attention mechanism and support vector machine

Visual attention mechanism can segment a image into salient and non-salient regions, which is convenient for people to recognize and retrieve the image. In this part, the visual attention mechanism and support vector machine will be combined to complete the annotation of images. This section will detail the whole process of the method.

### 4.1. Image pre-processing

First, the HC algorithm is used to process the image, dividing the image into salient regions and non-salient regions (Segmentation process is shown in 3). After the image is divided, the image regions are extracted. The original image is divided into two images according to the threshold value in the divided image. One is the saliency object graph, in which the salient region is preserved and the non-salient region is filled with white space. The other is the non-salient object, in which the non-salient areas are preserved, and the salient areas are filled with white space. Then we use a colored pattern appearance model (CPAM) to extract the features of the two images separately [31].

The CPAM model divides the region of the image to be extracted into 4 × 4 regions, and then extracts the Chromatic Spatial Pattern Histogram (CSPH) and the Achromatic Spatial Pattern Histogram (ASPH) for each small region. The CSPH and ASPH in all small regions are combined to form a feature vector representing the entire image. In this paper, the CSPH and ASPH are represented by a 64-dimensional eigenvector, and each image is represented by a 128-dimensional eigenvector. Supposing two images represented by the CPAM model are *x*_1_ and *x*_2_ respectively, the distance between two images can be calculated by formula (5).

$$d(x_{1},x_{2})=\sum_{\forall i}\frac{\left|ASPH_{1}(i)-ASPH_{2}(i)\right|}{1+ASPH_{1}(i)+ASPH_{2}(i)}+\sum_{\forall j}\frac{\left|CSPH_{1}(i)-CSPH_{2}(i)\right|}{1+CSPH_{1}(i)+CSPH_{2}(i)}$$ (5)

where |⋅| represents the absolute value; *CSPH*(*i*) represents the *ith* component of the image *CSPH* vector; *ASPH*(*j*) represents the *jth* component of the image *ASPH* vector.

After the feature is extracted from both the salient and the non-salient regions of the image, each image is represented by two 128-dimensional eigenvectors, namely a salient region feature vector and a non-salient region feature vector. The two eigenvectors are then merged into one eigenvector.

A detailed explanation will be helpful. Because the significance of salient region and non-salient region is different, that is, salient region is more important than non-salient region, it is necessary to give more weight to salient region feature vector to reflect its importance. The size of the salient areas in the image is usually much smaller than those of the non-salient areas. The number of significant area pixels *num*_*S* and the number of non-significant area pixels *num*_*N* in the image are respectively calculated. Let the weight of the significant region eigenvector be $u_{1}=\frac{1}{num\_S}$, and the weight of the non-salient region eigenvector be $u_{2}=\frac{1}{num\_N}$. Because usually *num*_*S* < *num*_*N*, so *u*_1_ > *u*_2_. If the salient area of an image is larger than the non-salient area, the values of *u*_1_ and *u*_2_ are swapped to ensure that the weights corresponding to salient area eigenvectors are always larger than the weights corresponding to non-salient area eigenvectors. Finally, the fused eigenvectors are normalized. Supposing the salient regional eigenvector of the image is *x*_1_, the non-salient region eigenvectors is *x*_2_, the weighted average and the normalized eigenvectors are *x*.

$$x=\frac{u_{1}x_{1}+u_{2}x_{2}}{u_{1}+u_{2}}$$ (6)

When the unknown image is marked, the feature vector *x* is used to represent the image.

### 4.2. Image model training and annotating

After obtaining the feature vectors of the salient and non-salient regions of an image, the known image is used to train by SVM. This paper specifically uses the support vector data description (SVDD) algorithm [32]. However, considering the literature [9] (our previous study), we used PSO to optimize the SVDD to improve the accuracy of the algorithm. Originally used to solve one-class classification problems, the SVDD has been later extended to solve multiple classification problems. So, we named the algorithm PVSVDD(P refers to PSO;V refers to Visual Attention Mechanism; SVDD refers to SVDD). SVDD differs from other SVM methods, in that it does not need negative sample data in training data, because it is classified by building a hypersphere that can surround all positive sample points as much as possible. SVDD constructs a hypersphere for each category of data, significantly reducing the complexity of solving problems and increasing computational efficiency.

Mathematically, the SVDD is a training set *T* = {(*x*_1_, *y*_1_), (*x*_2_, *y*_2_), …, (*x*_*l*_, *y*_*l*_)} ∈(*R*^*n*^ × *y*)^*l*^, *x*_*i*_ ∈ *R*^*n*^, *y*_*i*_ ∈ *y* = {1, 2, …, *K*}, *i* = 1, 2, …, *l*. For each class, a hypersphere that encloses all the data points of the training samples is created, defining the class *m* hypersphere (*a*_*m*_, *r*_*m*_), where *a*_*m*_ is the hypersphere center and *r*_*m*_ is the hypersphere radius. The goal is a problem solution that includes as many points as possible for all training samples while minimizing the radius of the hypersphere. Similar to an SVM, it also allows the sample points to fall outside the hypersphere, with a relaxation variable introduced to obtain the mathematical model of the SVDD problem.

$$\text{min}\;r_{m}^{2}+C_{m}\sum_{i:y_{i}=m}\xi_{i}$$ (7)

$$s.t.\;||x_{i}-a_{m}|{|}^{2}\leq r_{m}^{2}+\xi_{i}$$ (8)

$$\xi_{i}\geq 0,\forall y_{i}=m$$ (9)

where *C*_*m*_(custom constants) is the penalty factor.

When solving a problem, the original problem is transformed into a dual problem.

$$\underset{\alpha }{\text{max}}\sum_{i:y_{i}=m}\alpha_{i}K(x_{i}\cdot x_{j})-\sum_{i:y_{i}=m}\sum_{j:y_{j}=m}\alpha_{i}\alpha_{j}K(x_{i}\cdot x_{j})$$ (10)

$$s.t.\sum_{i:y_{i}=m}\alpha_{i}=1,0\leq \alpha_{i}\leq C,\forall i:y_{i}=m$$

*K*(*x*_*i*_ · *x*_*j*_) is a kernel function. In this paper, the kernel function is as follows:

$$K(x_{i},x_{j})=e^{-\frac{d(x_{i},x_{j})}{h}}$$ (11)

where *d*(*x*_*i*_, *x*_*j*_) is defined by formula (5), and *h* is the dimension of the image feature vector. For a point *x*, its distance from the center of the ball is:

$$f(x)=||x-a_{m}|{|}^{2}=K(x,x)-2\sum_{j=1}^{n}\alpha_{j}K(x,x_{j})+\sum_{i=1}^{n}\sum_{j=1}^{n}\alpha_{i}\alpha_{j}K(x_{i},x_{j})$$ (12)

When the point *x* is located outside the ball, there is deformation:

$$\sum_{j=1}^{n}\alpha_{j}K(x,x_{j})<\frac{1}{2}(1+\sum_{i=1}^{n}\sum_{j=1}^{n}\alpha_{i}\alpha_{j}K(x_{i},x_{j})-r^{2})$$ (13)

Similarly, when point *x* is located in the ball boundary or inside, there is deformation:

$$\sum_{j=1}^{n}\alpha_{j}K(x,x_{j})\geq \frac{1}{2}(1+\sum_{i=1}^{n}\sum_{j=1}^{n}\alpha_{i}\alpha_{j}K(x_{i},x_{j})-r^{2})$$ (14)

The kernel density estimation function is introduced for subsequent computation.

$$p(x)=\sum_{i=1}^{n}\omega_{i}\varphi (x,x_{i})$$ (15)

where *φ*(*x*, *x*_*i*_) is a window function and *ω*_*i*_ is the weight, where $\sum_{i=1}^{n}\omega_{i}=1$. In this paper, let

$$\varphi (x,x_{i})=\frac{1}{n}e^{-\frac{d(x_{i},x_{j})}{n}}$$ (16)

Finally, we get the probability relationship between the *mth* image and the unknown image by formulas (13)–(16).

$$p(x|m)=\sum_{i=1}^{n}\alpha_{i}K(x,x_{i})$$ (17)

Formula (17) indicates the probability of the image to be marked for the *mth* class image.

After training, the support vector and the corresponding multipliers for each type of image are obtained. The probability relationship between the image and each type of image can be calculated by formula (17). *p*(*w*_*i*_ | *I*) represents the probability that the annotation word of unlabeled image *I* is *w*_*i*_. Derived from the Bayesian formula:

$$p(w_{i}|I)=\frac{p(w_{i})p(I|w_{i})}{p(I)}$$ (18)

where *p*(*w*_*i*_ | *I*) is the probability of *w*_*i*_, which can be obtained from *p*(*w*_*i*_) = *n*_*i*_ / *n*, where *n*_*i*_ represents the number of image categories that contain the word *w*_*i*_ and *n* representing the total number of image categories; *p*(*I*) represents the probability of image *I*, which is fixed for a given image, so the value is not calculated uniformly in the calculation; *p*(*I* | *w*_*i*_) represents the probability of generating *I* from *w*_*i*_. In this paper, because the model *m* of each type image previously trained using SVDD is related to the corresponding keyword *w*_*i*_, so *p*(*I* | *w*_*i*_) = *p*(*x* | *m*_*i*_). Thus, we can calculate the probability of each word as the unknown image tagging the word, and then take the first four with the highest probability as the annotation words of the image.

### 4.3. Relationship between the annotation words

There are also some relationships between the marked words. For example, if there is a keyword "sky", "white clouds" is also likely to appear. The probability relationship between words is defined as follows. The probability relationship between all words is dynamically updated before each image is annotated, and the probability of each word annotation image is calculated to improve the annotation result.

Supposing the image collection is as follows:

$$T=\{I_{1},I_{2},\ldots ,I_{N}\}$$ (19)

where *I*_*i*_, *i* = 1, 2, …, *N* means that the image collection is divided into *N* classes. Supposing the annotation word set is as follows:

$$W=\{w_{1},w_{2},\ldots ,w_{M}\}$$ (20)

If *I*(*w*_*i*_) represents a collection of keywords *w*_*i*_, then the relationship between keywords *w*_*i*_ and *w*_*j*_ can be expressed as follows:

$$p(w_{i}|w_{j})=\frac{I(w_{i})\cap I(w_{j})}{I(w_{j})}$$ (21)

$$p(w_{j}|w_{i})=\frac{I(w_{i})\cap I(w_{j})}{I(w_{i})}$$ (22)

In summary, supposing that a set of annotation words of image *I* is *V*, and all annotation word sets are *U*. If *V* = ∅, The probability of annotating image *I* of all words in set *U* will be calculated.

$$p(w_{i}|I)=\frac{p(w_{i})p(I|w_{i})}{p(I)}$$ (23)

We take the words with the largest probability as the first tagging words. If *V* ≠ ∅, the probability of all the annotation words labeled images *I* in set *U* − *V*.

$$p(w_{i}|I,w_{1,}w_{2},\ldots w_{M})=\frac{p(w_{i})p(I|w_{i})\prod_{t=1}^{M}p(w_{t}|w_{i})}{p(I)p(w_{1},\ldots ,w_{M}|I)}$$ (24)

Therefore, the annotation of the image is given by formula (23) and (24). The probability between words changes due to the frequency of the labeled words. Each time a new image is added, the probability relationship between words changes due to the frequency of the marked words. Every time the image is marked, the probability between all the words is updated.

### 4.4. Determining the salient words

The determination of salient words takes place according to the procedure given here. After deriving the global annotation words *w*_*i*_(*i* = 1, 2, 3, 4) of the image, the following steps are performed for each word: First, find all the image categories *M*_*ik*_ containing the word. Then initialize all the distance *d*_*ik*_ = 0 and calculate the distance *d*_*ik*_ between the salient region eigenvector of the image to be marked and the salient model of image category *M*_*ik*_. At the conclusion, only the *d*_*ik*_ values corresponding to the words which are included in the marked image and belong to the salient region are greater than 0. The smallest *d*_*ik*_ is chosen from all the distance values, and the probability of the annotation word as a salient word for the image is calculated. Finally, the two words with the highest probability are used as salient words for the image, with the remaining two used as non-obvious words. All are output in descending order of probability.

### 4.5. Algorithm process

The algorithm process follows here steps:

1. Divide the annotated images in the image library and extract the salient areas and non-salient areas.
2. Extract the features from the salient regions and the non-salient regions separately to obtain the corresponding eigenvectors, finally obtaining the eigenvectors that finally represent the whole image by the weighted average of the two.
3. Use the PSO to optimize the SVDD, and the optimized SVDD to train the whole image and salient region eigenvectors. The whole image annotation model and the salient region annotation model are obtained respectively.
4. Place the weighted eigenvector of the image to be annotated into the whole image annotation model image and calculate the overall annotation of the image by combining the relationships between words.
5. Combine the annotation from the analysis of the salient area of the image and the words from the analysis of the whole image and output them firstly. The remaining annotation words are output as non-salient words after the salient words.

This last processing step is possible for two reasons. First, the pre-processing stage uses the visual attention mechanism to divide the image region into salient and non-salient regions with weighted feature vectors representing the image. Second, the training model stage distinguishes the salient and non-salient regions again. Therefore, in the final annotation result, the annotation words of the two parts can be distinguished, with the annotation words corresponding to the salient regions output first to improve image annotation result.

In image retrieval, user searches usually reflect an interest in the salient areas of the corresponding images. If an image annotation method that does not distinguish tag importance is applied to the image retrieval, the system returns all images with tagging words matching the user query, even if the tagging words are of low importance for the image. For example, if a user gives a keyword airplane, some of the images returned by the system to the user are images including an airplane. However, some images may contain an airplane in the picture background, while the user’s intention is to find airplanes as the main subject of the image. If the algorithm proposed in this paper is applied to image retrieval, the above problem can be solved, so as to improve the user search experience.

**Fig 1:**
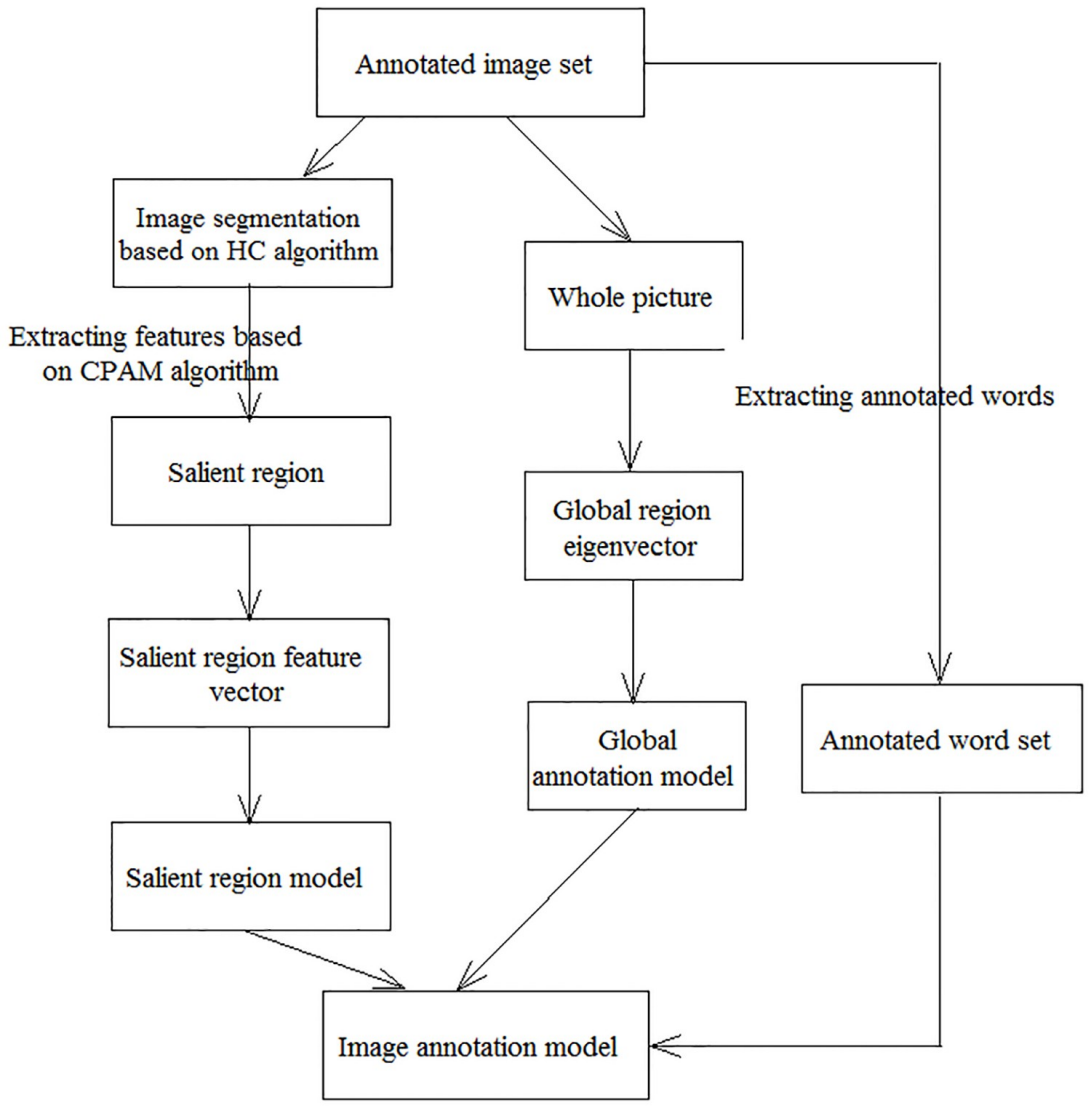
Training process.

**Fig 2:**
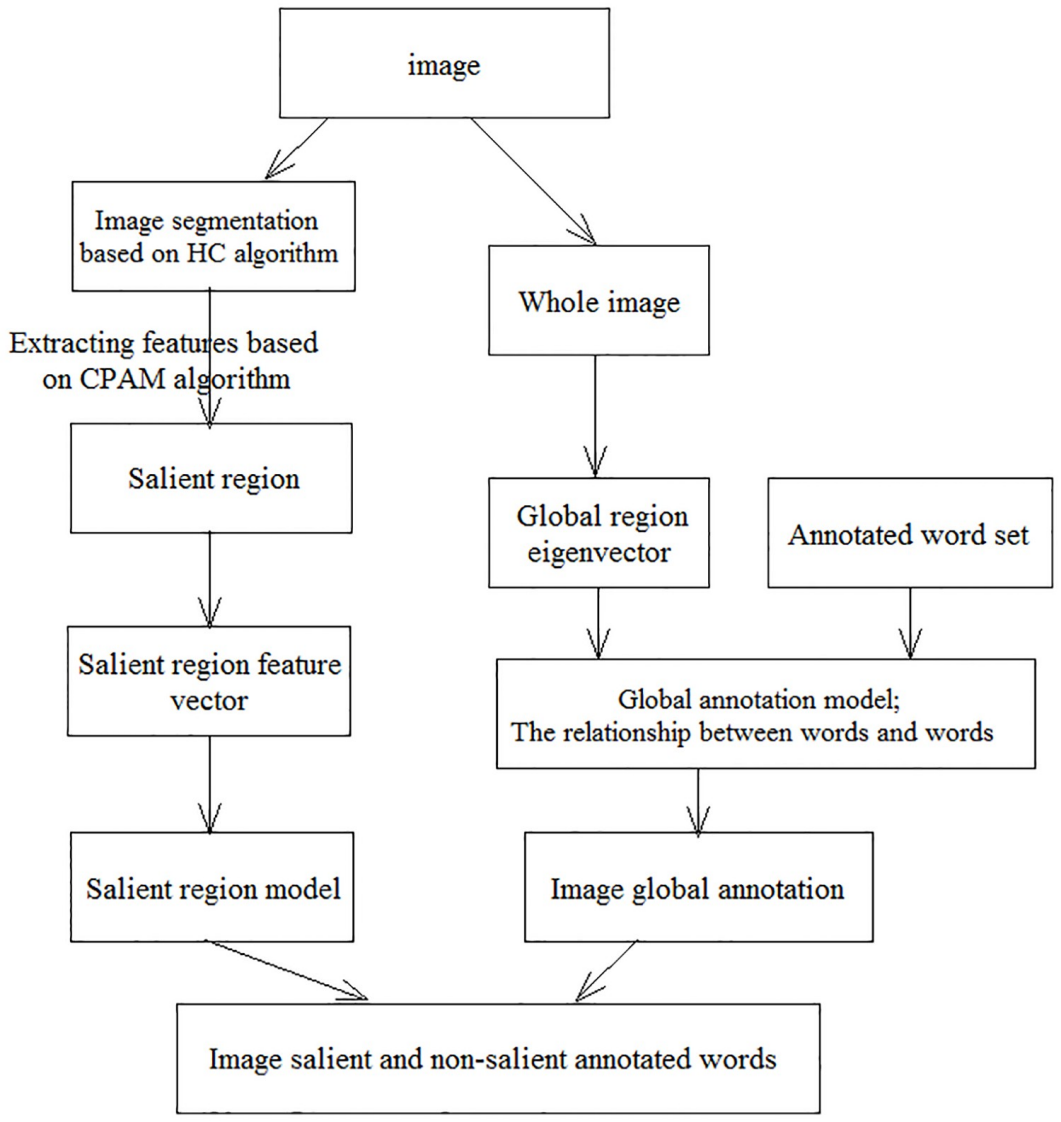
Annotation process.

In order to show the training and tagging process in detail, we give Fig 1(training process) and Fig 2(annotation process).

## 5. Experimental results

### 5.1. Experimental data sets and experimental environment

Our experiment used the Corel-5K image set [32] containing 5000 images divided into 50 categories and with 10 similar images in each category. In the experiment, 3 to 6 the initial tagging words were provided for each category, with a total number of 63 tagging words. The programming environment was MATLAB. The computer configuration is as follows: 2.60 GHz with 2.0GB RAM. A Gaussian kernel function was used in the SVDD algorithm, with *σ* = 1 / *N*, and penalty factor is *C* = 1 / *N*. We used *N* = 128 as the dimension of the image feature vector.

### 5.2. Performance comparison with other algorithms

**Fig 3:**
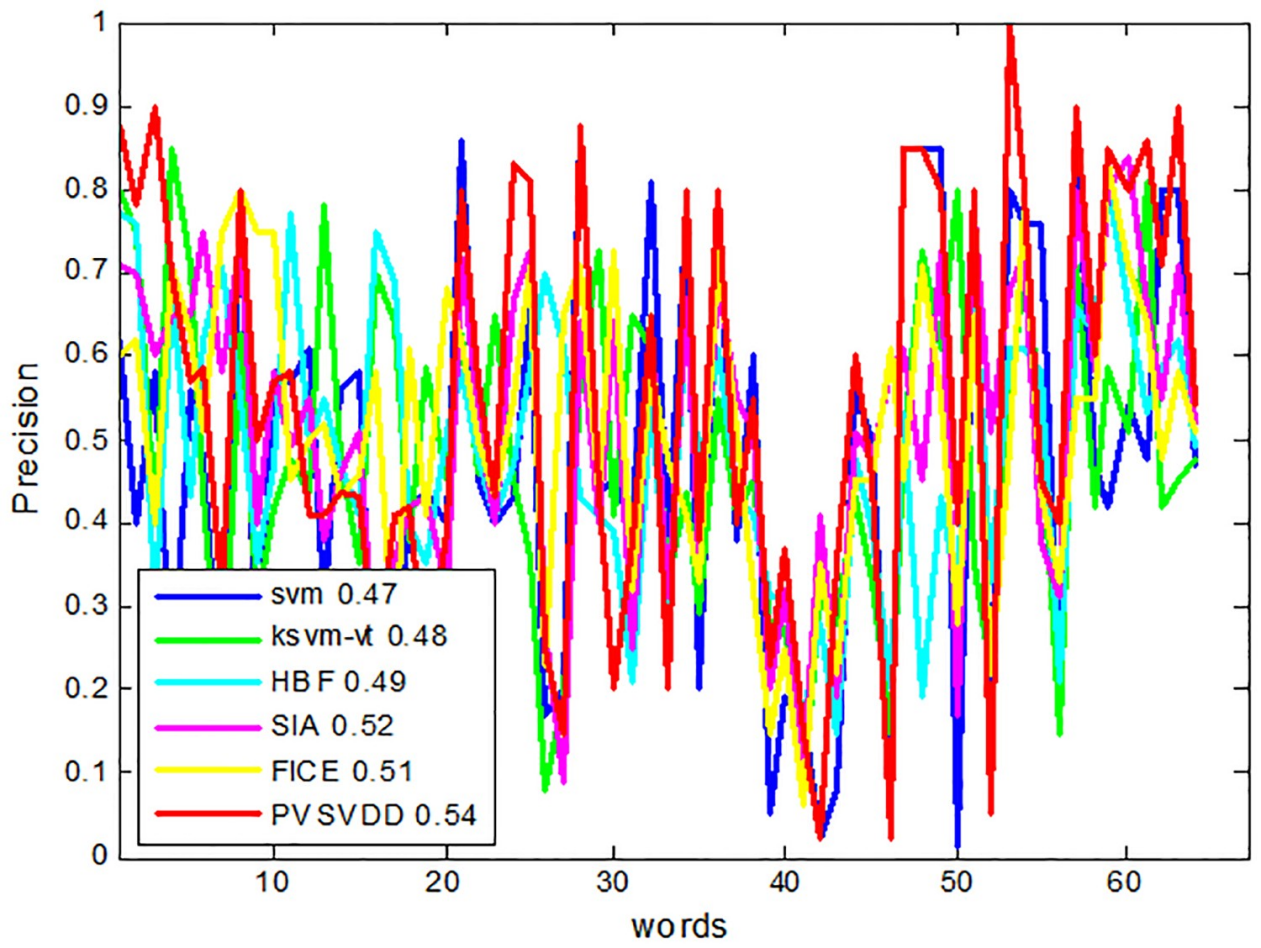
Comparison of precision of six algorithms.

**Fig 4:**
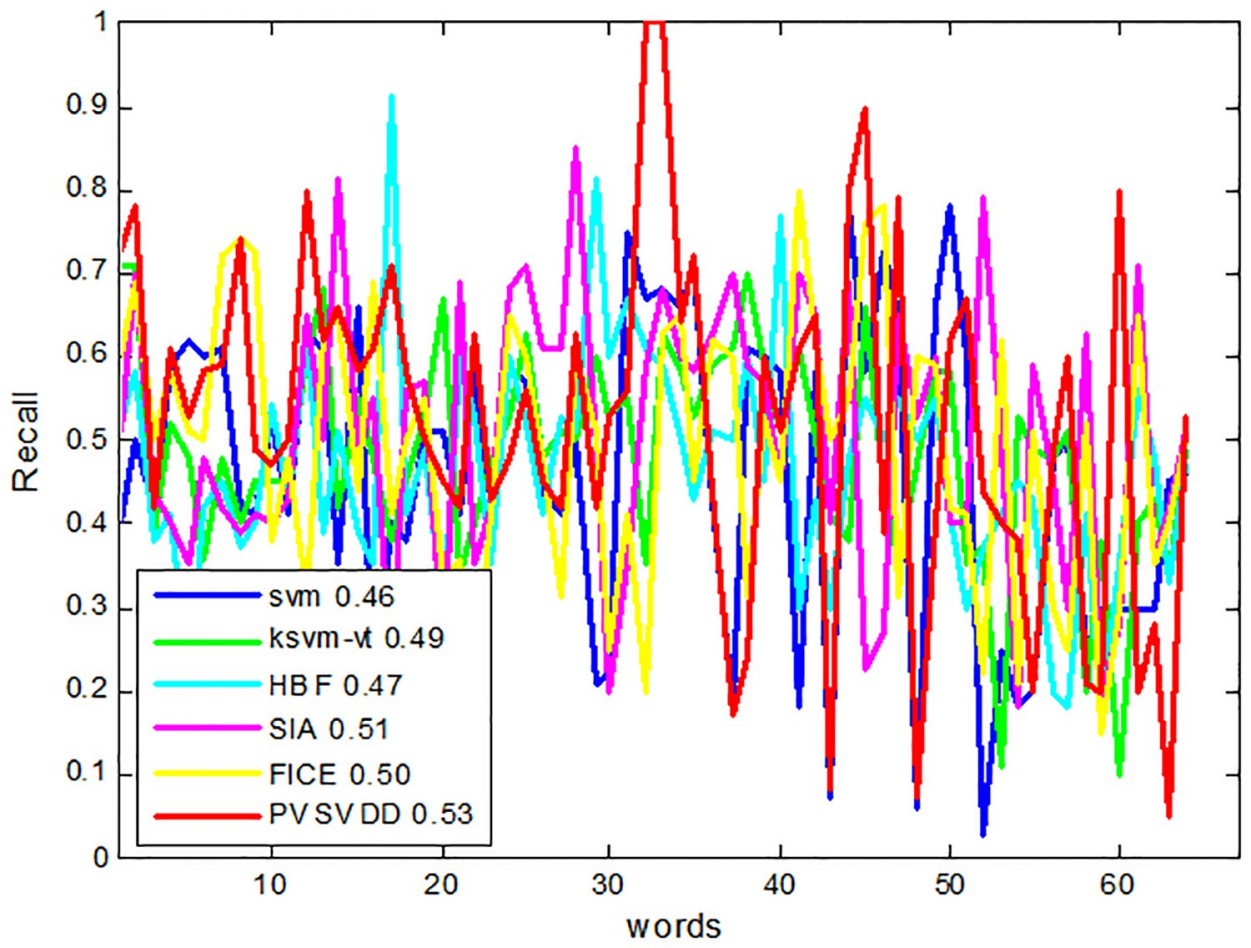
Comparison of recall of six algorithms.

To evaluate the performance of the proposed algorithm (named PVSVDD), the results were measured by recall and precision. We compared PVSVDD with five other related algorithms, SVM, KSVM-VT[13], HBF[11], SIA[28] and FICE[22]. We compared the recall and precision of the six algorithms for 63 annotation words. The last result reflects the average recall and precision of all the annotated words. The results are shown in Figs 3 and 4. Although the performance with some individual words is poor, the PVSVDD algorithm is better than the other five algorithms in general.

### 5.3. Image annotation

**Fig 5:**
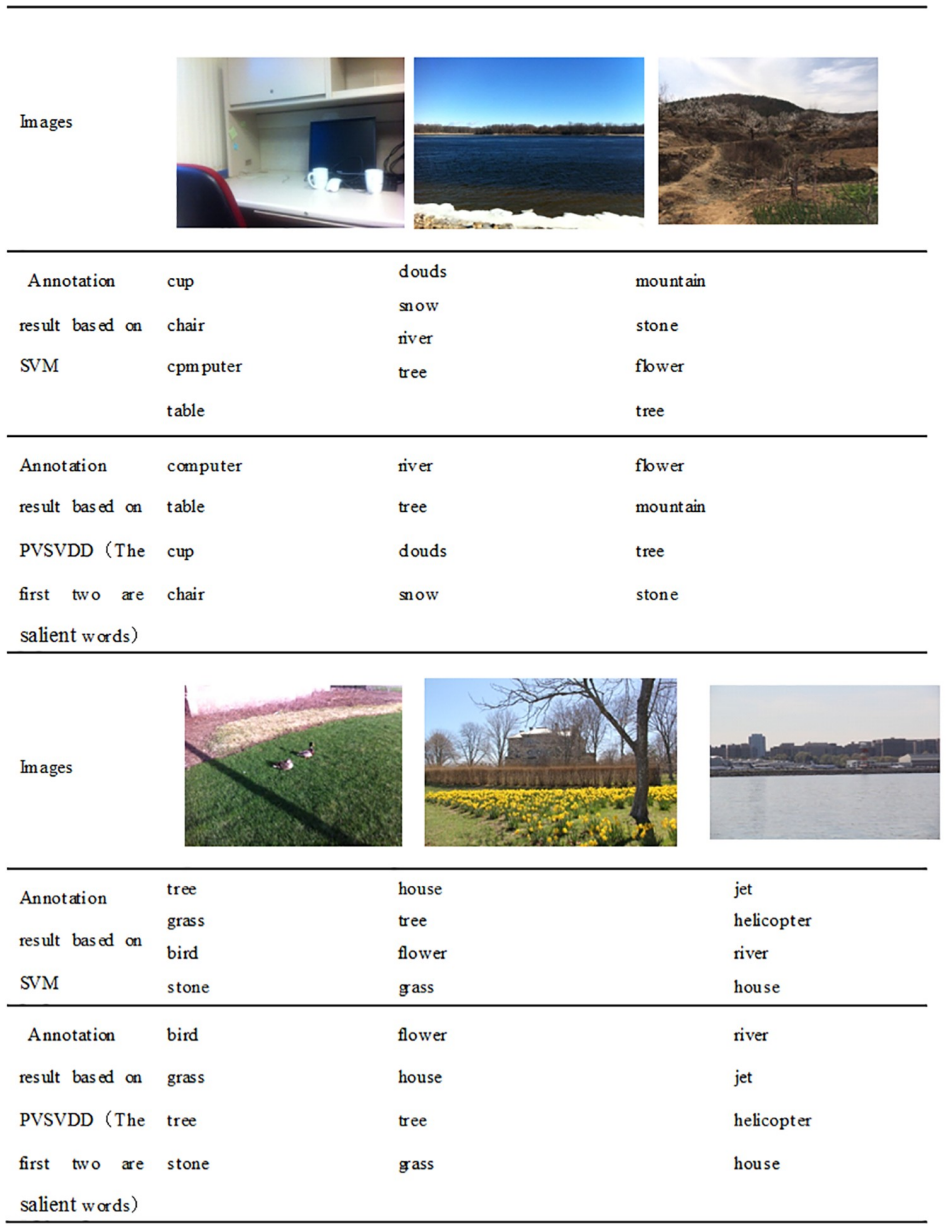
Example of annotation results. Reprinted from Qian Song under a CC BY license, with permission from Qian Song, original copyright Qian Song 2017.

Fig 5 shows the annotation results of several test images based on SVM and PVSVDD. Each image had four annotation words. The first two are salient words.

**Fig 6:**
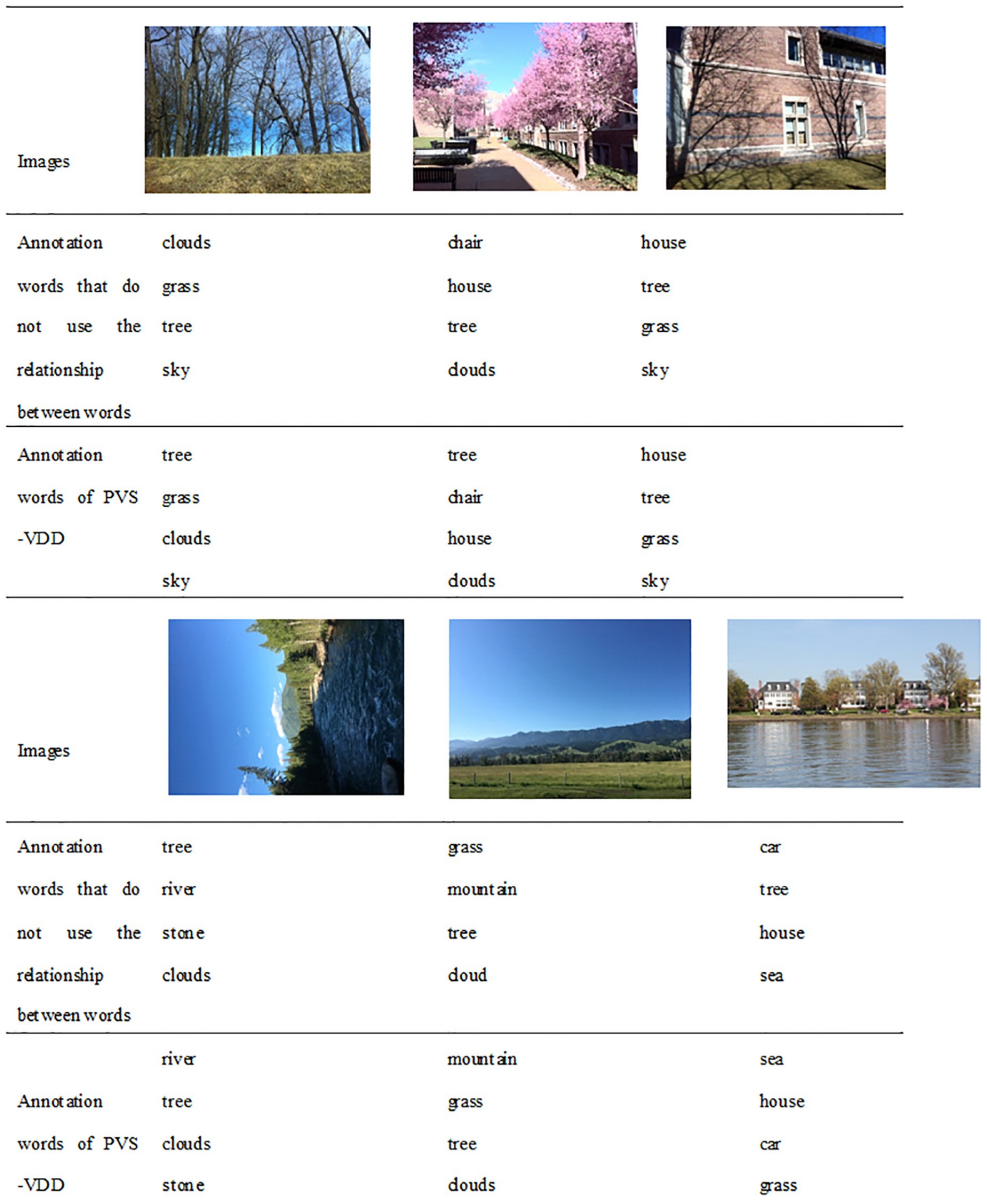
Comparison of using and not using relations of words. Reprinted from Qian Song under a CC BY license, with permission from Qian Song, original copyright Qian Song 2017.

When giving image annotation words, the algorithm considers the relationship between words, as described in section 4.3. Before marking each image, the probability relationship between words is updated. For example, if an image is labeled sun, sky, river, clouds, the frequencies of these four words is increased by one, changing the probabilities associated with these four words. As the image library is continuously updated, the probability of all words and other words is also continuously updated. The final image annotation results are affected as a result. Fig 6 shows the comparison of using and not using relations of words. The first two are salient words.

In this paper, the visual attention mechanism is fused into the image an notation process, so that the salient areas in the image can be labeled out first. This is often ignored in past annotations. As can be seen from the above two tables, the results of annotation using the PVSVDD algorithm show the salient areas of the image very well. The annotated words in front are marked the salient regions of the image. These salient regions are the contents that best reflect the content of the image, and are also needed for people to search. The final result can not only reflect the content of the image more accurately, but also improve the retrieval performance of the image.

## 6. Conclusion

Automatic image annotation not only has the efficiency of text-based image retrieval but also achieves the accuracy of content-based image retrieval. With it, users can find desired images by using keywords. However, until now, almost all the image annotation algorithms extract image features by extracting them without distinguishing the importance of different regions of an image, using the undifferentiated image as training for the annotation process. However, since the keyword given by the user is intended to represent a particular region of interest within the image, if the region of interest of the image can be extracted before annotation, the annotation results can be effectively improved. This leads to better image retrieval.

This paper explores these problems. Image preprocessing extracts salient regions of the image with the addition of the visual attention mechanism. A CPAM algorithm performs feature extraction on salient and non-salient regions, producing the corresponding eigenvectors. After weighting the eigenvectors of the salient regions of the image, the non-salient region eigenvectors are fused to obtain the final eigenvectors of the whole image. The SVDD algorithm based on PSO optimization is used to train and corresponding annotation models are obtained. Finally, the algorithm combines marked words and non-prominent marked words to produce the annotation results.
